# Effects of a Dark Septate Fungal Endophyte on the Growth and Physiological Response of Seedlings to Drought in an Epiphytic Orchid

**DOI:** 10.3389/fmicb.2022.961172

**Published:** 2022-07-08

**Authors:** Na Liu, Hans Jacquemyn, Qiang Liu, Shi-Cheng Shao, Gang Ding, Xiaoke Xing

**Affiliations:** ^1^Key Laboratory of Bioactive Substances and Resources Utilization of Chinese Herbal Medicine, Ministry of Education, Institute of Medicinal Plant Development, Chinese Academy of Medical Sciences and Peking Union Medical College, Beijing, China; ^2^Department of Biology, Plant Conservation and Population Biology, Katholieke Universiteit Leuven, Leuven, Belgium; ^3^Department of Ecological and Environmental Engineering, Yunnan Forestry Technological College, Kunming, China; ^4^Xishuangbanna Tropical Botanical Garden, Chinese Academy of Sciences, Mengla, China

**Keywords:** *Coelogyne viscosa*, orchids, root endophytes, dark septate endophytes (DSE), drought

## Abstract

Dark septate endophytes (DSE) are a group of facultative biotrophic root-colonizing fungi that live within a plant for a part of their life cycle without causing any apparent, overt negative effects. These fungi have been found in >600 different plant species, including orchids. Although the precise ecological functions of dark septate fungal endophytes are not yet well understood, there is increasing evidence that they enhance host growth and nutrient acquisition, and improve the plant’s ability to tolerate biotic and abiotic stresses. In this research, we tested the effects of a DSE isolated from the roots of the epiphytic orchid *Coelogyne viscosa* on the growth and drought tolerance of orchid seedlings. Our results showed that addition of DSE inoculum significantly enhanced biomass of seedlings and increased the activities of drought resistance related enzymes and the accumulation of osmoregulatory substances. These results suggest that DSE can fulfill important ecological functions in stressful environments and potentially play an important role in the life cycle of epiphytic orchids.

## Introduction

Endophytic fungi are fungi that reside mostly within plant tissues and grow within roots, stems, or leaves, without causing symptoms of disease (Rodriguez et al., 2009; Hardoim et al., 2015). Although endophytic fungi are known as important components of plant micro-ecosystems (Cordovez et al., 2019), their ecological function is not fully clear. There is increasing evidence that endophytes and plants often engage in a mutualistic relationship, with the endophyte enhancing host growth and nutrient acquisition, improving the plant’s ability to tolerate abiotic stresses, such as drought or salinity, and decreasing biotic stresses by enhancing plant resistance to insects, pathogens, and herbivores (Hardoim et al., 2015; Bamisile et al., 2018; Moraga, 2020). In exchange plants provide habitats and nutrients for the endophytic fungi (Latz et al., 2018).

One important group of fungal endophytes are dark septate endophytes (DSE), a group of anamorphic and root-inhabiting fungi, with dark septate or hyaline hyphae colonizing plant roots (Jumpponen and Trappe, 1998; Addy et al., 2005). DSE are widely distributed and are often the dominant fungal groups in plants growing in harsh habitats, such as Arctic, Antarctic, and alpine habitats, heavily polluted environments, arid ecosystems, and saline fields (Barrow, 2003; Mandyam and Jumpponen, 2005). In these harsh habitats, it has been demonstrated that DSE play a non-negligible role in promoting plant growth and development and mitigating biotic and abiotic stresses (Knapp et al., 2015; Qin et al., 2017; Chen et al., 2018). For example, the DSE *Phialophora mustea* has been shown to enhance metal tolerance and promote plant growth in tomato (Zhu et al., 2018). *Phialophora* sp. and *Leptosphaeria* sp. were shown to enhance antioxidase activity and drought tolerance in *Hedysarum scoparium* (Li et al., 2019).

In recent years, DSE have also been occasionally detected in the roots of orchids (Yuan et al., 2009; Ma et al., 2015; Novotná et al., 2017; Wang et al., 2021; Selosse et al., 2022). Orchids are known for their characteristic mycorrhizal symbiosis. Because an individual orchid produces thousands of tiny dust-like seeds, they have become fully reliant on typical orchid mycorrhizal fungi (OMF) to induce germination and subsequent establishment of seedlings. Besides, most adult orchids retain mycorrhizal associations as well (Rasmussen and Rasmussen, 2010) and these fungi have been recognized as an important factor influencing the distribution and abundance of orchid populations (McCormick and Jacquemyn, 2014; Pecoraro et al., 2018) and ecological adaptation of orchids (Herrera et al., 2018; Li et al., 2021). However, there is mounting evidence that fungi other than typical OMF reside in the roots of orchids, including DSE (Selosse et al., 2022), but to what extent DSE contribute to the ecology of orchids remains largely unclear.

Previous research has shown that several endophytic fungi can fulfill similar functions as typical OMF and contribute to seed germination and nutrient adsorption (Zhao et al., 2014), promote plant growth (Zhang et al., 2013), and induce pathogen resistance (Ma et al., 2015). In addition, it has been reported that endophytic fungi are able to enhance drought resistance (Kuldau and Bacon, 2008). As such, these fungi may fulfill an important role in the life cycle of epiphytic orchids, which by nature grow on the surface of tree barks and have limited access to water, especially during prolonged periods of drought in the dry season. However, whether DSE colonizing orchid roots are able to induce physiological changes that enhance drought resistance in orchids is still unclear.

In this study, we have isolated a DSE fungal strain from the roots of the epiphytic orchid *Coelogyne viscosa* growing on tree barks in tropic forest in Xishuangbanna, Southwest China. Previous research has shown that this orchid commonly associates with OMF of the Tulasnellaceae, Ceratobasidiaceae, and Sebacinaceae, and few other Basidiomycota members, such as Marasmiaceae, Thelephoraceae, Ganodermataceae, and unknown Basidiomycota (Xing et al., 2015), but presence of DSE in this epiphytic orchid was hitherto unknown. In order to understand the potential ecological functions of the isolated DSE fungus, we aimed at answering the following questions: (1) Does inoculation of orchid seedlings with this DSE affect growth of seedlings? (2) Does inoculation of the DSE induce physiological changes that enhance drought resistance of this epiphytic orchid? To answer these questions, sterile seedlings of *C. viscosa* were inoculated with DSE inoculum and subsequent seedling growth, enzyme activities and concentrations of osmoregulatory substances were compared between inoculated and non-inoculated control plants.

## Materials and Methods

### Orchid Species and Sampling

*Coelogyne viscosa* is an epiphytic orchid that grows in clusters on tree barks or rock surfaces. The species is widely distributed in northeastern India, Burma, southern China, Laos, Vietnam, Thailand, and peninsular Malaysia, where it grows in the evergreen lowland and montane forests at elevations between 700 and 1000 m a.s.l. The flowering period of *C. viscosa* ranges from September to November, and individual plants produce 2–4 flowers (Chen and Dudley, 2009).

This study was conducted in the Menglun sub-reserve (21°41′ N–101°25′ E), one of the five sub-reserves of the national nature reserve established in Xishuangbanna, Yunnan province, China. Ten individual plants were randomly selected and four root fragments (3–5 cm) were collected from each individual plant. Slight yellowish or opaque roots of *C. viscosa* were selected with surface being cleaned three times with sterile water to minimize the detection of substrate fungi. Roots were stored at 4°C for isolation of mycorrhizal associates. In addition, three mature capsules of *C. viscosa* were collected, and the capsules were surface sterilized and kept in plastic tubes at 4°C for sowing.

### Isolation and Identification of Fungal Associates

Root treatment and fungal isolation were performed according to Zhu et al. (2008). The collected roots were cut into 3-cm-long fragments, surface sterilized for 30 s in 75% ethanol, 3 min in 1% sodium hypochlorite solution, and 30 s in 75% ethanol, and washed six times in sterile water. Root hairs, epidermis, velamen, and other attachments were peeled off with a needle and forceps. Segments were scraped and teased apart. This released individual pelotons from the cortex cells. Pelotons were collected in a 60 mm sterile Petri dish containing 10 mL sterile distilled water. Then Petri dishes with pelotons were placed under a microscope at low power to observe the pelotons suspended in water. A 2.5 μL solution containing individual pelotons was absorbed onto 1 cm^2^ potato dextrose agar (PDA) with Streptomycin sulfate (100 μg/mL) and potassium Penicillin G (100 μg/mL) and cultured in a 25°C dark growth chamber. Visible hyphae emerged on the media within 1–2 weeks. The tips of the hyphae growing from the pelotons were cut and transferred to PDA several times for purification.

Genomic DNA was extracted from fungal isolates using the CTAB Plant Genome DNA Rapid Extraction kit provided by Beijing Aidlab Biotech Co., Ltd., Beijing, China according to the manufacturer’s instructions. For fungal identification, sequence amplification was performed using the universal primers ITS-1 and ITS-4 (White et al., 1990). All of the PCR reactions were carried out in a thermal cycler (BIO-RAD, America). The 25 μL PCR reaction system contained 12.5 μL Taq PCR Master (2×), 1 μL (5 μM) of each primer, 2 μL DNA template, and the remaining system was replenished to 25 μL with ddH2O. The PCR program was as follows: 95°C for 5 min, 35 cycles at 95°C for 45 s, 50°C for 30 s, and 72°C for 1 min with a final extension of 72°C for 10 min. The quality of extracted DNA was checked by 1% agarose gel electrophoresis and spectrophotometry (optical density at 260/280 nm ratio). Then the PCR solutions with single and bright bands were sent to the Taihe Biotechnology Co. Ltd. Beijing, China. for bi-directional DNA sequencing. For taxonomic identification, sequencing results were compared with known sequences from GenBank available on the National Center for Biotechnology Information (NCBI) website for a BLAST alignment.

### Germination and Growth of Seedlings

Three mature capsules of *C. viscosa* were sterilized following methods outlined in Dutra et al. (2009). Uncracked capsules were transferred in 75% alcohol for 1 min and 0.10% mercury chloride for 10 min, and washed six times with sterile water. Subsequently, the capsules were put on sterile filter paper for water absorption, and air-dried to remove the surface moisture. All operations were performed on a super-clean bench.

Next, the sterilized capsules were split and seeds were mixed thoroughly. Around 100 seeds were sown on 1/2 MS medium (Li and Li, 2017) in 30 Petri dishes and sealed in a 25°C constant dark chamber. The Petri dishes were transferred to a growth chamber (25°C, 3000 lux, light/dark = 12/12 h) after the embryos began to expand (about 2 weeks). Protocorms started to differentiate roots and leaves after 3 months of culturing. At that time, 5 seedlings were put in a cluster and transferred to one tissue culture bottle. In total, 120 bottles of seedlings were produced and cultured for another 8 months. Then 60 bottles of seedlings with well-developed roots and about 4 cm tall were used for subsequent experiments.

### Preparation of Dark Septate Endophytes-Inoculated Seedlings

Substrates used for seedling cultivation were prepared as follows: pine bark was crushed into small pieces (0.5–1 cm in diameter), soaked in water for 2 days for full water absorption and mixed with crumpled dry sawdust and chestnut leaves at a volume ratio of 1:1:1. Water was added to the substrate to obtain a relative water content of 80%. Then the mixed substrate was put into 60 culture bottles (60 g/bottle) and sterilized thoroughly in an autoclave at 121°C for 180 min.

Dark septate endophytes inocula were prepared by aseptically growing fungal isolates in Petri dishes with PDA medium. When the fungal colony reached 6 cm in diameter, the fungal cultures were used as inocula. The cultivated seedlings were carefully removed from tissue culture bottles, fresh weight was determined and subsequently seedlings were transferred to the substrate in cultivation bottles. For each bottle, a cluster of five seedlings was transferred, and a total of 60 bottles was used. Meanwhile, five plugs (5 mm) excised from the edge of an actively growing DSE colony were inoculated close to the roots of *C. viscosa* seedlings. Thirty bottles of seedlings were inoculated, while the other 30 were added with plugs excised from the sterile medium. All the bottles were sealed with sterilized gas-permeable sealing films. The inoculation processes were carried out on a super-clean bench. Then the seedlings were kept in a growth chamber with a 12 h light/12 h dark photoperiod, 25°C, and 60% air relative humidity. The hyphae were visible in the substrate a week later. All seedlings were cultured for 100 days to establish symbiosis. To investigate how inoculation with DSE affected growth of seedlings, fresh weight of seedlings was determined for inoculated and non-inoculated groups (*n* = 10 clusters of seedlings per treatment). The seedlings were carefully removed from the culture substrate and subsequently washed with running water, drained with filter paper and finally weighed to the nearest mg.

### Physiological Response to Artificial Drought

To assess how inoculation with DSE affected the physiological response of seedlings that were exposed to drought, the gas-permeable sealing film was removed from the cultivation bottles to mimic a natural drought. Twenty bottles with inoculated seedlings and 20 bottles with non-inoculated seedlings (control) were exposed to air at 25°C, 60% relative air humidity and 3000 lux light intensity (light/dark = 12/12 h). The experiment lasted for 12 days. Leaves were sampled at day 0, 3, 6, 9, and 12 for the measurement of physiological indexes. At each sampling timepoint, four bottles of seedlings were randomly selected from inoculated and non-inoculated groups, and leaves collected from each group were pooled. The sampled leaves were immediately wrapped in tinfoil and placed in liquid nitrogen, and then transferred to the –80°C refrigerator for storage. The culture bottles were weighed while sampling to record changes in relative water content of the substrate.

### Physiological Indexes Measurement

Crude enzyme extract was obtained by adding 0.2M PBS buffer (pH = 7.0) pre-cooled at 4°C at a ratio of 1 mL buffer per 0.1 g leaves according to the net weight of leaves. Subsequently, the mixture was grinded at a low temperature. After centrifugation at 4°C and 4000 rpm for 10 min, the supernatant was collected and stored at 4°C for 1–2 days for the measurement of peroxidase (POD), superoxide dismutase (SOD), and catalase (CAT) activities, and the concentrations of soluble proteins, sugars, and proline (PRO). For each determination, three technical replicates were used.

The POD and CAT activities were determined by a POD Assay Kit (colorimetric method) and a CAT Assay Kit provided by Nanjing Jiancheng Bioengineering Institute. The SOD activity and soluble proteins were measured by a Total SOD Assay Kit with NBT and a Bradford Protein Assay Kit provided by Shanghai Beyotime Biotechnology Co., Ltd. Shanghai, China. The assays were performed in general accordance to the instructions provided by the manufacturer. Before the SOD determination, the crude enzyme extract needed to be diluted eightfold to fortyfold.

The soluble sugars and PRO in the control and fungus-treated groups were determined by the Plant Soluble Sugar Content Assay Kit and PRO Content Assay Kit provided by Beijing Box Biotechnology Technology Co., Ltd. Beijing, China, respectively. For the determination of soluble sugars, the tested sample solution was configured according to a ratio of 0.05 g tissue to 0.5 mL distilled water. For the determination of PRO, the tested sample solution was configured according to a ratio of 0.05 g tissue to 0.5 mL extract from the kit. The rest of the steps were performed with reference to the manufacturer’s instructions.

### Data Analysis

To assess the taxonomic position of the isolated DSE, a Maximum likelihood (ML) tree was constructed. The ITS sequence obtained in this study and reference sequences (closely similar sequences and published DSE sequences retrieved from GenBank) were aligned in Clustal X version 2.0 (Larkin et al., 2007). The best-fit model for the dataset was identified using the Akaike Information Criterion implemented in jModel Test2 (Darriba et al., 2012). Evolutionary model K2 + G was identified as the best model. The ML phylogeny was constructed with MEGA 7.0 and clade support was estimated using a non-parametric bootstrap analysis with 1000 pseudo-replicated datasets.

To investigate whether physiological indexes differed between seedlings inoculated with HB3-1 and controls, a two-way ANOVA was used with inoculation treatment, culture time and their interaction included as fixed factors and physiological indexes as dependent variables. In addition, to assess how fungal inoculation affected the fresh weight of seedlings and the physiological indicators measured at the given days, paired and unpaired *t*-tests were used. Fresh weight, CAT, SOD, and POD activities, the concentration of soluble proteins, sugars, and PRO were used as dependent variables, whereas fungal treatment (inoculated versus non-inoculated plants) as independent variable. All analyses were performed using the statistical software GraphPad Prism version 7.0 for Windows (GraphPad Software, San Diego, CA, United States).

## Results

### Isolation and Identification of Dark Septate Endophytes

**FIGURE 1:**
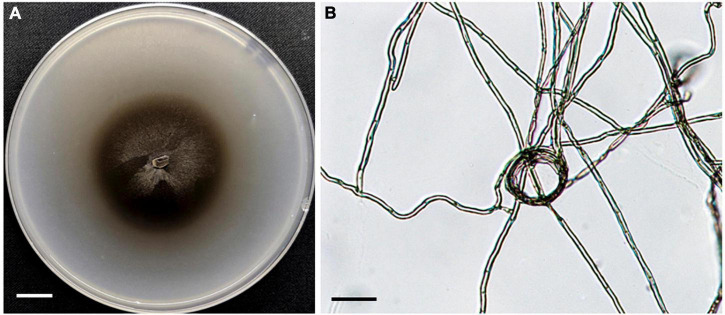
Morphology of fungal strain HB3-1. **(A)** Colony of HB3-1 on potato dextrose agar (PDA). **(B)** Mycelial morphology of HB3-1. The septate hyphae are visible. Scale bar, Panel **(A)** = 1 cm, Panel **(B)** = 20 μm.

**FIGURE 2:**
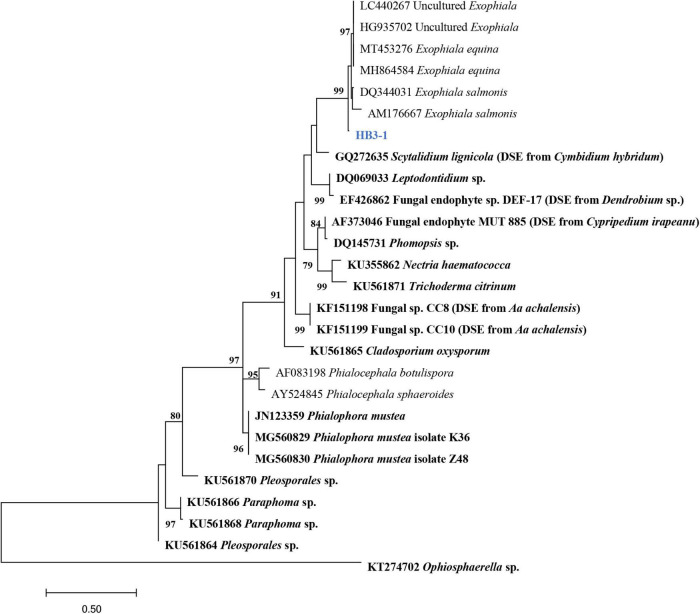
Maximum-likelihood tree constructed using rDNA-ITS sequences of HB3-1 generated in this study and reference sequences downloaded from GenBank. Known dark septate endophytes (DSE) are marked in bold. DSE detected in orchids (Girlanda et al., 2002; Hou and Guo, 2009; Valdés et al., 2011; Sebastián et al., 2014; Zhao et al., 2014) were marked by the orchid names in brackets. The values of the bootstrap frequencies (BP > 70%) are shown beside the nodes.

In total, 35 fungal isolates were retrieved by picking out pelotons directly from the cortex cells of root segments of *C. viscosa*. Combining morphological characteristics with ITS sequences, the 35 fungal isolates were identified as 7 fungal strains, four of them belonging to Ascomycota and three to Basidiomycota, respectively (Table 1). One fungal strain (HB3-1) had dark septate hyphae (Figure 1) and showed a 95% sequence similarity with *Exophiala* species [*E. equina* (MT4573276), Uncultured *Exophiala* (LC440267), etc.]. The sequence of HB3-1 was submitted to GenBank under the accession number OM327422. Based on the ITS sequences of HB3-1 and reference sequences of known DSE isolated from other plant species, we constructed a ML tree, and the result showed that HB3-1 clustered well with sequences of other *Exophiala* fungi from the same clade (Figure 2).

**TABLE 1:** Seven fungal strains isolated from roots of *Coelogyne viscosa.*

Fungal isolate	Closest matches in GenBank (accession numbers)	BLAST match sequence Max ident (%)	Proposed identification	Number of pelotons*
hy-1	Uncultured Tulasnellaceae (JF691394)	97.10	Tulasnellaceae sp.	4
hy-111	*Tulasnella* sp. (MK651838)	98.58	*Tulasnella* sp.	5
HBS1	*Flammulina velutipes* (MH469686)	96.97	*Flammulina* sp.	8
HB3-1	*Exophiala equina* (MT4573276)	95.20	*Exophiala* sp.	4
C-11	*Diaporthe phaseolorum* (KT964565)	97.35	*Diaporthe* sp.	7
C-22	*Acremonium charticola* (MK312365)	99.79	*Acremonium* sp.	4
cvl-1	*Gliomastix polychrome* (AB540566)	97.45	*Gliomastix* sp.	3

*Number of pelotons from which each fungal strain was recovered.

Colonies of strain HB3-1 grown on PDA were flat and fluffy, had an even edge and varied in color from dark to black-brown (Figure 1). There were few aerial hyphae around the inoculum. Colony thickness decreased gradually from the center to the edge, and colonies produced dark brown pigmentation on PDA medium. Colonies grew slowly, only reaching 6 cm in diameter after 4 months of culturing under 22 ± 2°C in darkness. The mycelium had branched, septate, light brown hyphae (2–6 μm in diameter). No sexual or asexual spores were observed.

### Inoculation and Verification of Dark Septate Endophytes Colonization

**FIGURE 3:**
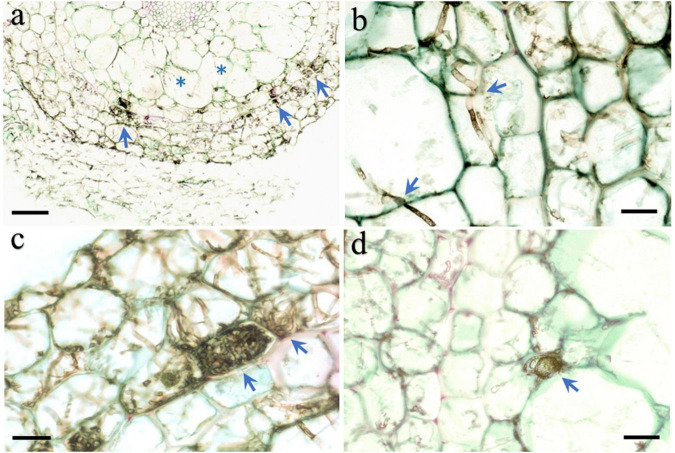
Morphological characteristics of HB3-1 colonization in roots of *Coelogyne viscosa* seedlings. **(A)** The root surface is surrounded by intense hyphae and the hyphae invaded into velamen (arrow) and cortex (asterisk) cells. **(B)** Hypha in the velamen and cortex cells penetrate through cell wall and colonized neighbor cells (arrow), Septate in hyphae can be observed. **(C)** HB3-1 form peloton-like structures within the velamen cells (arrow). **(D)** Microsclerotium formed at the intercellular space (arrow). Scale bars: Panel **(A)** = 80 μm; Panels **(B–D)** = 20 μm.

Microscopic observation of the roots of seedlings 100 days after they were inoculated with strain HB3-1 showed substantial fungal colonization. The colonized hyphae had a dark-brown moniliform structure. HB3-1 formed atypical pelotons in the velamen and exodermis cells (Figures 3A,B), and further extended inward and penetrated the cortical cells (Figure 3C). Furthermore, microsclerotia, a typical structure of DSE, were observed in the cortical intercellular space (Figure 3D). From these morphological observations, it was concluded that the observed strain showed all features characteristic of typical DSE and therefore had successfully colonized seedlings.

### Effect of Dark Septate Endophytes Inoculation on Fresh Weight of Seedlings

**FIGURE 4:**
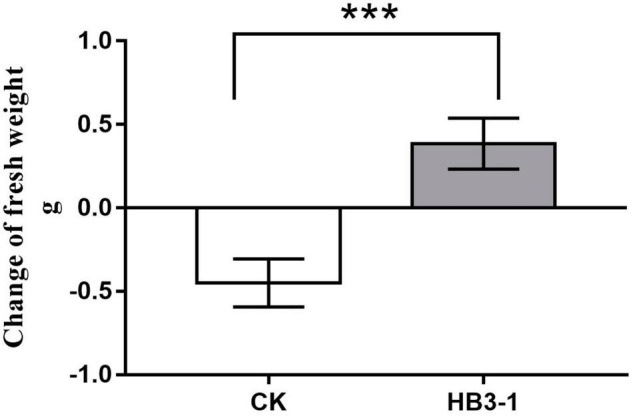
Change of fresh weight of seedlings inoculated with HB3-1 and the control group after 100 days of culture on cultivation substrate. *** means a difference that is significant at level α = 0.001.

HB3-1 had a significant effect on growth of *C. viscosa* seedlings. When seedlings of *C. viscosa* were transplanted from the nutrient-rich 1/2MS medium to the relatively infertile bark substrate, the growth rate of seedlings slowed down in both the treatment and control, due to their need to re-adapt to the new environment. One hundred days after inoculation, fresh weight of non-inoculated seedlings had reduced by 0.45 ± 0.07 g and was significantly lower (*t* = 6.27, *P* < 0.01) than before transplantation. Interestingly, the fresh weight of seedlings inoculated with HB3-1 had increased by 0.39 ± 0.08 g and was significantly higher than before transplantation (*t* = 5.04, *P* < 0.01) (Figure 4).

### Effect of Dark Septate Endophytes Inoculation on Physiological Indexes

Inoculation with HB3-1 significantly affected the physiological variables measured in seedlings of *C. viscosa*. Two-way ANOVA revealed that the activities of all investigated physiological indexes were significantly influenced by inoculation, culture time and their interactions, except for POD and soluble protein, which were not influenced by inoculation, and CAT, which was not influenced by culture time (Table 2).

**TABLE 2:** Effect of inoculation with HB3-1 (inoculation vs. control) and culture time on physiological indexes of *Coelogyne viscosa* seedlings.

Extracellular enzyme	Inoculation		Culture time		Inoculation × Culture time	
	*F*	*p*	*F*	*p*	*F*	*p*
SOD	216.126	**<0.001**	234.764	**<0.001**	148.273	**<0.001**
CAT	53.430	**<0.001**	1.845	0.160	9.903	**<0.001**
POD	0.789	0.835	11.936	**<0.001**	30.602	**<0.001**
Soluble protein	3.074	0.095	57.850	**<0.001**	51.708	**<0.001**
Soluble sugar	88.467	**<0.001**	340.993	**<0.001**	8.571	**<0.001**
Proline	54.638	**<0.001**	64.312	**<0.001**	43.873	**<0.001**

*Bold values are significant at P < 0.05.*

**FIGURE 5:**
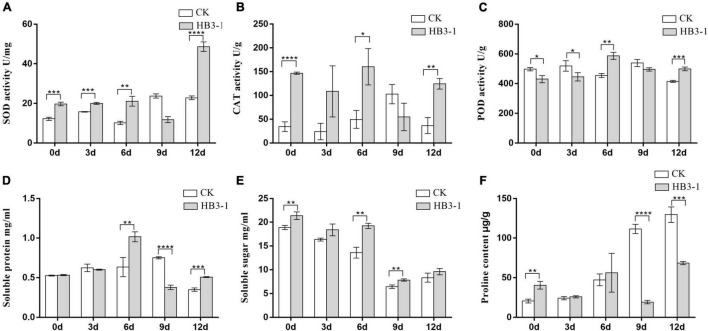
Differences in physiological variables between control (CK) seedlings and seedlings inoculated with dark septate fungal endophyte strain HB3-1. **(A)** Superoxide dismutase (SOD) activity. **(B)** Catalase (CAT) activity. **(C)** Peroxidase (POD) activity. **(D)** Soluble protein content. **(E)** Soluble sugar content. **(F)** Proline (PRO) content. * *P* < 0.05, ** 0.001 ≤ *P* < 0.01, *** 0.0001 ≤ *P* < 0.001, **** *P* < 0.0001.

Superoxide dismutase activity in the seedlings inoculated with HB3-1 was significantly (*P* < 0.01) higher than that in the control group during most of the experiment, and the difference in expression level increased as drought progressed, reaching a maximum value at day 12 (Figure 5A). Similarly, a significantly higher CAT activity (*P* < 0.05) was detected in seedlings inoculated with HB3-1 than in control seedlings (day 0, 6, and 12) (Figure 5B). POD activity was slightly higher in control seedlings than in seedlings inoculated with HB3-1 in the beginning of the experiment (day 0 and 3) (*P* < 0.05), but became significantly lower (*P* < 0.01) at the end of the experiment (day 6 and 12) (Figure 5C).

The soluble protein content in control seedlings reached its peak at day 9 (0.751 ± 0.008 mg/mL), while in the HB3-1 treatment group this peak was already detected at day 6 (1.015 ± 0.036 mg/mL) and it was significantly higher (*P* < 0.01) than in the control (Figure 5D). The soluble sugar content in both HB3-1-treated and control groups decreased during the experiment, but it was always significantly higher in the HB3-1 group than in the group control (*P* < 0.01) (Figure 5E). PRO content did not differ significantly between the HB3-1-treated seedlings and control seedlings in the beginning of the experiment, but it was significantly higher in the control group than in HB3-1-treated group at the end of the experiment (day 9 and 12, *P* < 0.001) (Figure 5F).

## Discussion

In this study, a DSE fungus was isolated from the roots of the epiphytic orchid *C. viscosa* and identified as *Exophiala*. Fungi from the genus *Exophiala* have been previously reported to live in symbiosis with many plants as DSE, promoting plant growth and development and improving plant resistance against biotic and abiotic stresses (Khan et al., 2011; Zhan et al., 2015; Zhang et al., 2017). Here, we investigated the potential ecological function of HB3-1 by re-inoculating the fungus to the roots of aseptically cultured seedlings of *C. viscosa*. The results showed that fresh weight of seedlings inoculated with HB3-1 significantly increased after they had been transplanted to a medium resembling natural conditions, indicating that colonization of the roots of *C. viscosa* may promote the growth and development of seedlings in natural environments. The increase in plant weight suggests that there is more organic matter to support the plant and to survive longer periods of high temperatures and drought that characterize the environments where this epiphytic orchid naturally occurs.

Our results further showed that seedlings in the HB3-1 treatment group had higher antioxidant enzyme levels than seedlings in the control group, suggesting a greater ability to eliminate reactive oxygen species (ROS) (Amiri et al., 2015). ROS are known to cause deterioration of lipids, proteins and nucleic acids in plants when extreme environmental changes occur, such as drought, salinity, or floods, etc., and act as the critical signaling molecules throughout the cell death pathway (Kapoor et al., 2019). At the same time, the higher concentrations of soluble proteins and glucose indicate an enhanced osmotic ability of seedlings inoculated with HB3-1, and suggest that seedlings are more conducive to maintain turgor pressure under drought (Liu et al., 2021). Since PRO is involved in several physiological processes such as regulating osmotic potential and redox reactions, affecting the transmission of energy and preventing the production of ROS (Sharma and Verslues, 2010; Azmat and Moin, 2019), accumulation of PRO is a common physiological response to various stresses. Our experiments showed that PRO levels were lower in the HB3-1-treated group than in the control group, suggesting that inoculated seedlings were less susceptible to drought stress than control seedlings.

Previous studies have already demonstrated the growth and anti-stress effect of DSE. It has been shown that DSE promote plant growth (Jumpponen et al., 1998; Jumpponen, 2001), but the mechanisms are not always clear. Narisawa et al. (2004) showed that DSE can provide nitrogen to the host plants. Similar results were obtained by Upson et al. (2009), who showed that DSE isolates increased growth of *Deschampsia antarctica* when the plants were supplied with an organic N source. However, no such effects were observed when plants were supplied with ammonium. Wu et al. (2021) used transcriptome sequencing of DSE-inoculated cherry and showed that DSE improved nitrate assimilation of sweet cherry through NO_3_^–^ transporters. There is also mounting evidence that plants inoculated with DSE display higher resistance to various forms of stress. Li et al. (2018), for example, reported that DSE promoted growth of *Ammopiptanthus mongolicus* under drought conditions, and *Exophiala pisciphila* improved the Cd tolerance of maize (He et al., 2017). The mechanism by which DSE enhance drought resistance is related to the characteristics of the fungus itself. DSEs are mostly melanin-rich fungi. Melanin and chitin can protect the host in adverse environments by increasing the rigidity of cell walls and reducing permeability (Addy et al., 2005). Barrow (2003) suggested that DSE acquire and transport carbon from the host to the root surface and form polysaccharide mucus on the root surface, thus helping plants to maintain the transport of water and nutrients in arid environments.

As an epiphytic orchid, *C. viscosa* grows in tree canopies to obtain light (Gravendeel et al., 2004), but at the same time they have less access to water (Chase, 1987). Seasonal water shortage has led to the evolution of a pseudobulb, a storage organ that preserves water for leaves during drought. Mycorrhizal fungi have been widely reported in the past years, but it is still unclear whether these symbiotic fungi play a role in enhancing stress resistance in the host plant. However, based on the results presented in this study, it is reasonable to believe that DSE play an active role in drought resistance in the roots of *C. viscosa*. Additionally, the results of this research further indicate that more attention should be paid to fungal endophytes such as DSE to elucidate their role in mediating ecological adaptation of epiphytic orchids to stressful environments.

## Conclusion

In this study, a DSE was isolated from the roots of *C. viscosa* collected in natural conditions by picking out pelotons residing in cortical cells, and reinoculation verified that this DSE could promote the growth of *C. viscosa* seedlings. Furthermore, our experiments showed that seedlings inoculated with DSE had higher levels of antioxidant enzyme activities, and more accumulation of osmotic regulating substances than seedlings from the control group. We therefore conclude that DSE have the potential to enhance drought resistance in epiphytic orchids and therefore play a more important role in the ecology of epiphytic orchids than previously assumed. However, the extent to which they are observed in nature and the molecular mechanisms taking place in the orchid-DSE interaction process remain to be elucidated.

## Data Availability Statement

The original contributions presented in this study are included in the article/supplementary material, further inquiries can be directed to the corresponding author.

## Author Contributions

XX and HJ designed the research and revised the manuscript. NL, QL, S-CS, and GD performed the research. NL analyzed the data. NL, XX, and HJ wrote the manuscript. All authors have read and agreed to the published version of the manuscript.

## Conflict of Interest

The authors declare that the research was conducted in the absence of any commercial or financial relationships that could be construed as a potential conflict of interest.

## Publisher’s Note

All claims expressed in this article are solely those of the authors and do not necessarily represent those of their affiliated organizations, or those of the publisher, the editors and the reviewers. Any product that may be evaluated in this article, or claim that may be made by its manufacturer, is not guaranteed or endorsed by the publisher.
